# Bridge to Life: Current Landscape of Temporary Mechanical Circulatory Support in Heart-Failure-Related Cardiogenic Shock

**DOI:** 10.3390/jcm13144120

**Published:** 2024-07-14

**Authors:** Panayotis K. Vlachakis, Panagiotis Theofilis, Ioannis Leontsinis, Maria Drakopoulou, Paschalis Karakasis, Evangelos Oikonomou, Christina Chrysohoou, Konstantinos Tsioufis, Dimitris Tousoulis

**Affiliations:** 1 1st Department of Cardiology, “Hippokration” General Hospital, National and Kapodistrian University of Athens, 11527 Athens, Greece; vlachakispanag@gmail.com (P.K.V.); giannisleontsinis@gmail.com (I.L.); mdrakopoulou@hotmail.com (M.D.); chrysohoou@usa.net (C.C.); kptsioufis@gmail.com (K.T.); drtousoulis@hotmail.com (D.T.); 2 2nd Department of Cardiology, “Hippokration” General Hospital, Aristotle University of Thessaloniki, 54642 Thessaloniki, Greece; pakar15@hotmail.com; 3 3rd Department of Cardiology, Thoracic Diseases General Hospital “Sotiria”, National and Kapodistrian University of Athens, 11527 Athens, Greece; boikono@gmail.com

**Keywords:** cardiogenic shock, heart failure, intra-aortic balloon pump, Impella, TandemHeart, extracorporeal membranous oxygenation

## Abstract

Acute heart failure (HF) presents a significant mortality burden, necessitating continuous therapeutic advancements. Temporary mechanical circulatory support (MCS) is crucial in managing cardiogenic shock (CS) secondary to acute HF, serving as a bridge to recovery or durable support. Currently, MCS options include the Intra-Aortic Balloon Pump (IABP), TandemHeart (TH), Impella, and Veno-Arterial Extracorporeal Membrane Oxygenation (VA-ECMO), each offering unique benefits and risks tailored to patient-specific factors and clinical scenarios. This review examines the clinical implications of recent advancements in temporary MCS, identifies knowledge gaps, and explores promising avenues for future research and clinical application. Understanding each device’s unique attributes is crucial for their efficient implementation in various clinical scenarios, ultimately advancing towards intelligent, personalized support strategies.

## 1. Introduction

**Figure 1:**
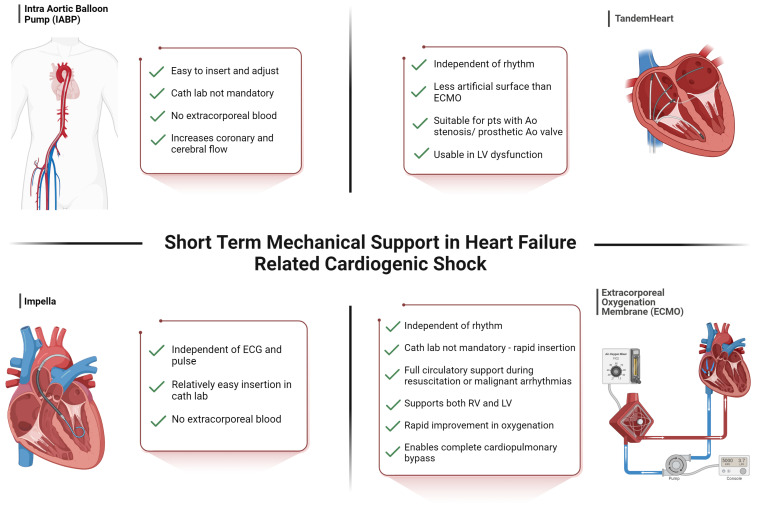
Advantages of short-term mechanical circulatory support in heart-failure-related cardiogenic shock. Created with BioRender.com. ECG: electrocardiogram, RV: right ventricle, LV: left ventricle.

Acute heart failure (HF) carries an unacceptably high mortality burden, necessitating continual advancements in therapeutic strategies to improve patient outcomes. Temporary mechanical circulatory support (MCS) has emerged as a crucial intervention in the management of cardiogenic shock complicating acute heart failure, providing a bridge to recovery or to a durable version of mechanical support. As the landscape of temporary MCS evolves, it becomes imperative to critically assess the current status on the field, examining the existing literature, identifying gaps in knowledge, and evaluating the clinical implications of recent advancements (Figure 1). This comprehensive review aims to explore the spectrum of temporary MCS devices, including their mechanisms of action, indications, and comparative effectiveness, with a keen focus on identifying the most promising avenues for future research and clinical application.

## 2. Temporary Mechanical Circulatory Support Devices

### 2.1. Intra-Aortic Balloon Pump

#### 2.1.1. Principle of Circuit

In previous decades, Intra-aortic Balloon Pump (IABP) has been one of the most widely used devices to improve hemodynamics in patients suffering from cardiogenic shock (CS). This popularity was driven by several features such as being a relatively simple and available at-bedside device with no obligatory need for fluoroscopy upon insertion, also with a notable low complication rate [1,2]. The device specifications include a cylindrical balloon mounted on a catheter, which is typically inserted into the aorta through the femoral artery [3]. Timed with the cardiac cycle, stimulated by either pressure waveform or electrocardiogram (ECG), inflation occurs during diastole, in a manner that peripheral and coronary perfusion is enhanced. Consequently, afterload reduction off-loads the left ventricle and cardiac output (CO) increases [3].

In the context of acute decompensated HF (ADHF), IABP deployment yields notable benefits through key pathophysiological mechanisms. IABP support modestly enhances CO, particularly beneficial in scenarios with disproportional left ventricle (LV) afterload elevation [4]. It effectively addresses systemic vascular resistance (SVR) escalation seen in severe LV dysfunction and malperfusion, reaching its peak in overt cardiogenic shock (CS) [5]. Counterpulsation with IABP contributes to the reduction in SVR within the initial 12 h, accompanied by heightened LV power indexes in ADHF [6]. In the same lines, IABP’s venting effect becomes more evident in occasions when afterload is further artificially increased by circulatory support such as the case of venoarterial extracorporeal membrane oxygenation (VA-ECMO) [7]. The prevalence of functional mitral regurgitation (MR) associated with worsening LV systolic dysfunction is sensitively addressed by IABP’s afterload reduction [8,9]. Importantly, the modulation of splanchnic blood flow by IABP may prove beneficial, especially in hypoperfused ADHF cases with moderate/severe kidney dysfunction, where a more negative fluid balance was reported compared to inotropes [10]. Finally, the combination of afterload reduction with myocardial perfusion improvement not only spares the dosage of energy-consuming inotropic agents but also ameliorates the arrhythmic substrate, providing not only mechanical but also electrical improvements [11].

#### 2.1.2. Contraindications—Considerations

The synchronization of balloon inflation and deflation with the cardiac cycle is imperative. Poor-quality ECG or cardiac arrythmias may lead to inaccurately timed balloon pumping, thereby nullifying hemodynamic benefits and intensifying LV workload [12]. Furthermore, the presence of more than mild aortic regurgitation constitutes one of the absolute contraindications for the implantation of IABP. This is attributed to the potential elevation of LV end-diastolic volume and pressure during balloon inflation, which increases afterload while myocardial perfusion becomes jeopardized [13]. Complications associated with the IABP may be divided into non-vascular and vascular events, with the latter posing the predominant risk in IABP utilization. The hazard of these complications increases proportionally with the diameter of the catheters and arterial sheaths [14,15].

#### 2.1.3. Clinical Evidence

Despite enthusiasm rooted in the pathophysiological premises of IABP, the disappointing findings related to short- and long-term survival in a post-acute myocardial infarction (MI) settings and/or in acute MI complicated by CS have significantly diminished its clinical utilization and the strength of recommendations [16,17,18]. It is noteworthy that while the utilization of IABP use decreased for MI-related CS, it has concurrently risen for other indications [19,20]. This aligns with the growing proportion of individuals presenting with CS due to ADHF, which presently constitutes the most prevalent etiology of CS in modern cardiac intensive care units [21]. This shift is also reflected in increased utilization of intensive care resources during ADHF hospitalization, indicating a rising proportion of critically ill patients with ADHF [22].

To date, the majority of data on the use of this device come from several observational studies on patients with ADHF (Table 1). Uli and colleagues conducted the only prospective randomized control trial with a small yet highly selected group, demonstrating that the use of IABP in 32 patients experiencing ADHF with low output yielded positive outcomes. Specifically, after 48 hours of treatment, IABP improved mixed-venous oxygen saturation (+17 [+9; +24)% vs. +5 [+2; +9)%, *p* < 0.001), increased cardiac power output (*p* = 0.004), lowered NT-proBNP levels (4.907 pg/mL vs. 8.772 pg/mL, *p* = 0.01), and reduced dyspnea severity scores (*p* = 0.02) compared to treatment with inotropes. Significantly, 30-day all-cause mortality exhibited a numerical decrease in the IABP group (23% vs. 44%, *p* = 0.25), while no serious adverse events related to IABP were reported during the study [23].

### 2.2. TandemHeart

#### 2.2.1. Principle of Circuit

TandemHeart (TH) (CardiacAssist Inc, Pittsburg, PA, USA) has emerged as a percutaneous ventricular assist device (VAD) designed to provide transient support during high-risk cardiac procedures and in cases of CS. TH provides superior hemodynamic support compared to IABP. However, the existing body of evidence supporting its application in high-risk populations is limited, primarily relying on case studies and observational data. The procedure’s inherent requirement for trans-septal cannulation renders TH the most invasive among the reviewed devices, limiting its clinical applicability to specialized centers with skilled interventional cardiologists proficient in trans-septal punctures.

During TH therapy, oxygenated blood is suctioned by the left atrium and pumped back into the arterial circulation bypassing the left ventricle. The procedural intricacies involve the insertion of a 21F drainage catheter into the left atrium (LA) via a trans-septal approach from the femoral vein. The centrifugal pump aspirates oxygenated blood from the LA, propelling it into the arterial circulation through a 15–17F return catheter. The meticulous positioning of the drainage catheter necessitates the expertise of an interventional cardiologist. Throughout TH support, blood flow redirection from the LA to the aorta results in diminished LV preload, filling pressures, workload, and myocardial oxygen demand. Its extra-corporeal continuous-flow centrifugal pump can provide flow rates up to 4 L/min at a maximum speed of 7500 rpm, while connection to an oxygenator may also assist in cases of impaired gas exchange. TH stands out as a favorable choice for MCS in cases of isolated LV failure. It obviates the necessity for an additional mechanism for LV unloading, a requirement often encountered when employing peripheral VA-ECMO.

#### 2.2.2. Contraindications—Considerations

Despite enhancing systemic circulation, TH’s unique feature of retrograde aortic blood flow increases afterload, potentially counteracting the myocardial protective benefits associated with left ventricular unloading. Contraindications include severe peripheral vascular disease, right or left atrial thrombi, ventricular septal defects (causing right-to-left shunting and hypoxia), inadequate right ventricular (RV) function, aortic regurgitation, and conditions precluding the use of unfractionated heparin. Possible complications, such as vascular injury, bleeding, distal limb ischemia, cardiac tamponade after trans-septal puncture, infection, catheter migration, stroke, and intracranial hemorrhage, necessitate vigilant management and consideration of alternative interventions in specific scenarios.

#### 2.2.3. Clinical Evidence

While most of the evidence with TH concerns high-risk cardiovascular interventions, there have been a few studies assessing its effectiveness and safety in the setting of advanced HF complicated by CS (Table 2). In a recent study of 55 patients undergoing TH implantation for CS (18 with ADHF), the intervention led to the amelioration of hemodynamic (cardiac index, pulmonary capillary wedge pressure) and laboratory indices (creatinine, lactate) within the first 24 h [31]. The mean duration of TH support was 5.2 days. Interestingly, the patients in this study were classified as bridge to LVAD and bridge to recovery prior to the device’s insertion, with those in the latter category facing a grim prognosis. Specifically, 23.8% in this group survived hospital discharge compared to 68.2% in those assigned to bridge to LVAD, while 30-day mortality was also higher in those on a bridge to recovery strategy. These findings highlight the importance of having an exit strategy with acceptable longer-term outcomes before TH implantation.

The study of Kar et al. also delved into the role of TH in 117 cases of severe refractory CS due to ischemic and nonischemic cardiomyopathy [32]. It is important to note that only five patients from this study had an acute myocardial infarction. The majority of the study’s participants were on IABP support prior to TH implantation, and approximately 50% were already receiving cardiopulmonary resuscitation (CPR) at the time of insertion (mean time from CPR onset to TH implantation 65 min). The mean duration of TH therapy was slightly longer (mean 5.8 days) but similarly improved hemodynamic (cardiac index, pulmonary capillary wedge pressure), clinical (urine output, systolic blood pressure), and laboratory variables (mixed venous oxygen saturation, lactate, creatinine). Fifty-five percent of patients with multi-organ failure deceased despite TH support. Adverse events were frequent, with the majority being bleeding around the cannula site, sepsis, and gastrointestinal bleeding. Thirty-day and six-month mortality rates were 43.8% and 50%, respectively, with those rates being higher in recipients of permanent LVAD/heart transplant.

### 2.3. Impella

#### 2.3.1. Principle of Circuit

The Impella device emerges as a pivotal short-term MCS option for patients facing CS. It is distinguished by an axial flow pump employing a non-pulsatile Archimedes-screw design to propel blood from the LV into the ascending aorta, operating in series with the LV [34]. The Impella device facilitates an increase in CO, cardiac power, and mean arterial pressure (MAP), along with a corresponding elevation in coronary and systemic perfusion pressures. The effective unloading of LV contributes to a reduction in LV end-diastolic pressure, resulting in decreased wall tension and, consequently, a reduction in myocardial oxygen demand.

It comes in three versions, namely, the 12F (Impella 2.5), 14F (Impella CP), and 21F (Impella 5.0) devices, offering flow rates of 2.5, 3.0, to 4.0, and 5.0 L/min, respectively. These devices are typically inserted through the femoral artery, either percutaneously (for 2.5 and CP) or with a surgical cutdown (for 5.0 and 5.5). The catheter features a flexible pigtail loop at its tip for stabilizing in the LV, reducing the risk of perforation. The various sizes and deployment methods cater to diverse clinical needs, and alternative access sites such as the subclavian artery have been reported. A clinical trial is currently examining the Impella expandable cardiac power (ECP) device, implantable through a smaller sheath (9F), capable of delivering flows up to 3.5 L/min (NCT04477603). The Impella 5.0 and 5.5, due to its size, necessitates a surgical cutdown for deployment via the axillary or femoral artery, potentially offering long-term support [35].

Unlike the IABP, the Impella operates independently of timing and does not require triggers from ECG rhythms or arterial pressure. It has been approved by the FDA for providing up to 6 h of partial circulatory support, while in Europe, the Impella 2.5 is sanctioned for use up to 5 days. This pump, similar to TH, offers stability during transient arrhythmias but poorly tolerates asystole and ventricular fibrillation [35]. Additionally, there is the option of the Impella RP, specifically designed for addressing right HF. Implanted via the femoral vein, the Impella RP provides support to the RV. However, its effectiveness may be influenced by factors such as the patient’s sedation or paralysis status and the filling of the RV, making the device susceptible to variations in blood volume and patient movement [36].

#### 2.3.2. Contraindications—Considerations

The utilization of the Impella device is contraindicated in individuals with significant aortic valve disease, a mechanical aortic valve, LV thrombus, severe peripheral arterial disease, or those unable to tolerate systemic anticoagulation [37]. Additionally, caution is warranted in patients with a known, pre-existing ventricular septal defect due to the potential exacerbation of right-to-left shunting and hypoxemia. Despite being less invasive than surgically implanted devices, the application of intracardiac micro-axial flow pumps, such as Impella, is not exempt from potential complications [35]. Reported issues encompass limb ischemia, bleeding, and various vascular injuries, including hematoma, pseudo-aneurysm, and arterial–venous fistula, emphasizing the necessity for meticulous patient selection and vigilant monitoring during device deployment [37]. Mechanical erythrocyte shearing may lead to hemolysis, a complication observed in a notable proportion of patients within the initial 24 h of use [38]. These considerations underscore the importance of a judicious approach to Impella utilization, taking into account both contraindications and potential complications.

#### 2.3.3. Clinical Evidence

The device’s ability to offer robust hemodynamic support positions it as a valuable tool in the armamentarium for managing advanced HF (Table 3). In a retrospective study of 58 inotrope-dependent patients with ADHF receiving Impella 5.0 as a bridge to decision, conducted from 2010–2015, survival to next therapy occurred in 67% of cases, with 1-year survival rates of 65% for those receiving durable MCS, 87% for heart transplants recipients, and 75% for those stabilized and weaned, demonstrating the potential efficacy of Impella as a bridge-to-decision strategy in this challenging population [39]. Regarding the expansion of Impella’s role as a bridge to transplant, a study investigated heart transplant outcomes with Impella 5.0 support. Sixteen advanced HF patients received Impella 5.5 before heart transplantation, showing improved renal function (median creatinine serum level decreased from 1.55 mg/dL to 1.25 mg/dL, *p* = 0.007), increased pulmonary artery pulsatility index (from 2.56 to 4.2 *p* = 0.048), and enhanced RV function (*p* = 0.003) during support; patients maintained these benefits post-transplantation, with all surviving without significant morbidity [40].

Another clinical scenario for using this device in patients with ADHF is those who undergo ventricular tachycardia (VT) ablation [45,46]. Initial studies suggested that the use of MCS leads to benefits in prolonged VT cases, enhancing mapping and ablation and resulting in increased VT terminations with radiofrequency ablation (*p* = 0.03) [46,47]. In a large non-randomized study comparing Impella (n = 109) to non-percutaneous LVAD (n = 85), no significant difference was found in VT termination success during ablation or freedom from VT recurrence in follow-up. Notably, patients receiving Impella support were in poorer health, characterized by lower LV ejection fractions (26 ± 10% vs. 39 ± 16%; *p* < 0.001), a higher incidence of New York Heart Association (NYHA) class ≥ III HF (51% vs. 25%; *p* < 0.001), and a more frequent occurrence of electrical storm (49% vs. 34%; *p* = 0.04) [43]. Consequently, researchers concluded that temporary MCS allowed sicker HF patients to achieve outcomes comparable to those in patients in better health conditions [43]. However, conflicting studies challenged this conclusion, casting uncertainty on the evidence supporting the benefits of temporary MCS in reducing VT recurrence and mortality following catheter ablation [48].

In terms of acute cardiogenic shock, the recently published Danish–German Cardiogenic Shock (DanGer Shock) trial demonstrated that the use of Impella compared to standard of care in more than 350 patients (Median LVEF ~ 25%) suffering from STEMI and cardiogenic shock led to lower risk of death from any cause at 180 days (45.8% vs. 58.5%, HR 0.74; 95% CI, 0.55 to 0.99; *p* = 0.04) [44]. However, the risk of adverse events, specifically the need for renal replacement therapy, was observed more frequently in those who randomized to MCS compared to those who were treated according to the standard of care (41.9% vs. 26.7%, RR 1.98; 95% CI, 1.27 to 3.09). Authors mentioned that this trial stands out from other contemporary randomized trials of mechanical circulatory support due to its more homogenous patient population. The enrollment criterion, which required a mandatory elevation in arterial lactate level without a cardiac arrest, identified patients with profound left ventricular failure and a high incidence of adverse events. This is evidenced by the substantial mortality observed beyond the 30-day follow-up [44].

### 2.4. Extracorporeal Membranous Oxygenation

#### 2.4.1. Principle of Circuit

Veno-arterial ECMO (VA-ECMO) is a contemporary MCS consisting of a centrifugal flow pump, a membrane oxygenator, and cannulation of a large vein and artery. It is indicated in patients with CS refractory to optimal medical therapy. For its insertion, an 18–28F sheath is commonly introduced into the venous side, while a 15–19F sheath is preferred for arterial cannulation. Central placement of VA-ECMO involves direct surgical right atrial cannulation (venous inflow) and ascending aorta cannulation (arterial outflow). Although this technique provides a better venous drainage with less concern for upper body hypoxia, it does not come without risks since it may lead to bleeding complications, mediastinitis, aortic dissection, and pulmonary thrombus formation, among others. According to a meta-analysis of 17 retrospective case series, bleeding complications, the need for continuous venovenous hemofiltration, and transfusions were more commonly seen with central cannulation, even though the in-hospital survival rates were comparable to those of peripheral VA-ECMO [49].

Peripheral insertion of VA-ECMO, which is the most common practice, can be either femorofemoral (inflow: femoral vein; outflow: femoral artery) to provide retrograde perfusion, or involve the upper extremity vascular system (inflow: internal jugular or subclavian vein; outflow: axillary, subclavian, or carotid artery), thus providing antegrade perfusion. Peripheral VA-ECMO insertion could be performed outside of the operating room, such as in the catheterization laboratory, the intensive care unit, the emergency room, or even out of hospital in the case of cardiac arrest.

The reported rates of successful weaning in the literature vary greatly. To ensure successful myocardial recovery that adequately supports end-organ perfusion and meets the body’s metabolic demands, clinical, hemodynamic, and echocardiographic data should align. Any metabolic disturbances or dysfunction in end-organs should be rectified or addressed through alternative means [50,51]. Pulmonary function should not be severely compromised, and maintaining pulmonary oxygenation with a PaO_2_/FiO_2_ greater than 200 on 0.21 FiO_2_ is advised. Transitioning to VV-ECMO is recommended for patients with a PaO_2_/FiO_2_ ratio lower than 100 [51]. In the absence of or with low doses of catecholamines and vasopressors, the baseline MAP should exceed 60 mmHg [50,51]. When attempting VA-ECMO weaning, it is common to use another form of temporary MCS, such as IABP or Impella. Many patients are successfully weaned from VA-ECMO with these devices still in place.

The most recent guidelines, put forth through a scientific statement by the American Heart Association (AHA) in 2022 [52], suggest the daily evaluation of cardiac function. The objective is to discontinue VA-ECMO as soon as patients exhibit improvement in the root cause of their CS, achieve intravascular euvolemia, and maintain hemodynamic stability with minimal intravenous support. The recommended approach involves a gradual reduction in support flow, typically decreasing in increments of 0.5 to 1 L/min until reaching a range of 1.5 to 2.0 L/min, signaling the appropriate time for decannulation. The standard frequency for this stepwise flow reduction is typically every 2 to 4 h.

#### 2.4.2. Contraindications—Considerations

Among VA-ECMO’s contraindications are an expected poor prognosis (limited life expectancy, irreversible multiorgan failure, neurological injury), significant aortic regurgitation, an inability to receive anticoagulation, advanced age, and cognitive impairment, among others. We should note that although age by itself should not be the most important determinant, age ≥80 years old is an independent predictor of mortality in an analysis of the Extracorporeal Life Support Organization Registry, while acute decompensated HF in the elderly constitutes another scenario associated with poor prognosis [53].

Specific complications should be stressed such as limb ischemia, which could be addressed by selecting the right cannula size based on ultrasonographic measurements, as well as by implementing a distal perfusion cannula to the limb. Moreover, Harlequin syndrome (also known as North–South Syndrome) is a VA-ECMO-specific complication where the recovery of LV function precedes the adequate lung gas exchange. The pumped oxygenated blood from ECMO meets the deoxygenated blood from the LV and, in this case, the pumped blood enters the distal portion of the aorta, resulting in coronary and cerebral hypoperfusion.

#### 2.4.3. Clinical Evidence

The use of ECMO has increased over time according to the Extracorporeal Life Support Organization Registry. Mastoris et al. documented that ECMO was used as a bridge to permanent LVAD or orthotopic heart transplantation in 1.7% of CS patients in 2010, with that number rising astonishingly to 22.2% in 2019 [54]. However, we lack clear evidence of its actual benefit in patients presenting with refractory CS in the setting of HF (Table 2). The Extracorporeal Membrane Oxygenation in the Therapy of Cardiogenic Shock (ECMO-CS) represents a major trial assessing VA-ECMO in CS, with a significant proportion (35.1%) of the 117 enrolled patients having a non-MI etiology of CS [33]. This multicenter study included rapidly deteriorating patients or with established CS who were randomized to undergo an early VA-ECMO placement or an initially conservative therapy, with death from any cause, resuscitated circulatory arrest, and implementation of another mechanical circulatory support device at 30 days being the primary composite outcome. Despite the absence of specific data on clinical parameters, therapy at randomization, and downstream VA-ECMO use for the conservative arm of this population, the incidence of the primary endpoint or all-cause mortality did not differ across the study arms for this subgroup.

While VA-ECMO is extremely efficient in restoring circulation and maintaining CO, it is ineffective in unloading the ventricle. This increased LV afterload in the setting of LV systolic dysfunction could lead to pulmonary edema. In such cases, LV unloading with mechanical venting to the LA will result in unloading of the LA to the venous cannula, maintaining a low filling pressure to the left heart. Other options of LV unloading are IABP or Impella. The analysis of the Extracorporeal Life Support Organization registry of 12734 patients receiving VA-ECMO for CS provided insights into the importance of mechanical LV unloading [55]. Of the involved subjects during a 10-year period, 2987 (25.6%) had chronic HF, with 31.5% of those receiving mechanical unloading (IABP: 60.6%, other percutaneous MCS: 39.4%). In-hospital mortality rates were 12% lower in this subgroup when using LV unloading (adjusted OR: 0.88%, 95% CI 0.73–1.06). Other findings of this study deserve to be mentioned, although specific information regarding the group of patients with HF as the CS etiology is not available. First, there was a higher rate of complications (gastrointestinal bleeding, cannula site bleeding, tamponade, hemolysis, ischemic stroke, acute kidney injury, need for renal replacement therapy) in the LV unloading arm. Early or even upfront implementation of mechanical unloading was associated with a lower incidence of acute kidney injury, but no difference in mortality rates. Another critical observation was the similar efficacy outcomes between IABP and other peripheral MCS for LV unloading paired with a better safety profile (lesser bleeding and acute kidney injury).

The combined use of VA-ECMO with Impella (ECPELLA) has received increased attention over the years. In a retrospective study by Patel et al. in 66 patients with refractory CS, mortality rates at 30 days were significantly lower in the ECPELLA group compared to VA-ECMO-only group, together with a lower need for inotropic support, suggesting the potential benefit of such a combined approach [56]. In a study of 686 patients with CS treated with ECMO with or without Impella-assisted LV unloading, the authors also noted lower mortality rates in the ECPELLA arm, at the cost of increasing complications [57]. The V-A ECMO AUTO Mode Registry’s (NCT05759377) assessment of the combination of those methods is expected to provide further evidence in this direction.

Other options for LV unloading in conjunction with VA-ECMO have been described. Atrial septostomy, either surgically or percutaneously performed, involves the creation of a left-to-right shunt aiming to alleviate ventricular workload by reducing preload and afterload [58]. Findings from the largest registry on atrial septostomy (223 patients) indicate that it is associated with notable complications (arrhythmias, tamponade, need for unplanned surgery) without procedural mortality, however [59]. Chronic lung disease and the emergency of the procedure are among the factors that may be linked with early mortality following the procedure [59]. Less studied options are pulmonary artery venting through jugular vein cannulation and LA venting [60,61].

## 3. Current State and Future Perspectives

The unprecedented advances in cardiovascular care have significantly transformed the practice of cardiology over the past four decades. The integration of primary preventive measures with effective pharmacotherapy, advanced devices, and corrective procedures for structural heart diseases has shifted the practice towards more proactive, durable, and minimally invasive therapies, exemplified by the success of Transcatheter Aortic Valve Implantation (TAVI). Additionally, heart transplantation has entered a new era, achieving average survival rates of good quality that now exceed a decade.

However, the bridge between stability and definitive treatment, such as transplantation, remains a topic of ongoing debate. Although this review does not focus on durable MCS, it is noteworthy that the HeartMate 3, the only currently available LVAD, has demonstrated success in supporting patients until their next stage, whether it be transplantation or not. In contrast, durable MCS for RV support has yet to show impressive results. In this review, we aim to evaluate whether different means of short-term MCS are ready to serve as a bridge to life for patients with advanced HF who left stability and entered the grey zone of early to refractory CS.

In 2018, the United Network for Organ Sharing (UNOS) revised their heart allocation policy, significantly impacting the prioritization of patients supported by temporary MCS devices. Under the new policy, these patients were assigned higher priority statuses, specifically statuses 1 or 2 [62]. As a result, the number of patients bridged with temporary MCS devices increased. Specifically, candidates with IABPs are assigned status 2, and the use of IABPs increased from 8.8% to 31.7% (*p* < 0.001) of transplant recipients since the policy change. Moreover, the number of transplanted patients bridged with VA-ECMO device increased more than 5-fold (*p* < 0.001) under the current system [63].

Current guidelines suggest that temporary MCS may be considered for selected patients experiencing CS (2022 AHA Level of Evidence B, 2021 ESC IIa, C) [64]. The European guidelines provide a class IIB recommendation for inotropic support, while the American guidelines offer a class IA recommendation, reflecting the reality in most institutions [65]. The utilization of temporary MCS varies significantly worldwide, with Mediterranean countries underutilizing these devices compared to Central and Northern Europe. This discrepancy is multifactorial, with guideline recommendations playing a less crucial role.

Until recently, the definition of CS was not universally standardized. Early recognition and stratification of CS patients into different risk levels are crucial for timely and appropriate treatment, improving prognosis [66]. Another important factor is the relatively weak current evidence on the survival benefits of temporary MCS. Studies such as IABP-SHOCK II, which compared IABP with other MCS devices like Impella, showed no significant differences in mortality. Randomized studies like EURO-SHOCK, ECMO-CS, and ECLS-SHOCK have shown mixed results, and ongoing trials like ANCHOR (NCT04184635) aim to provide further insights [33,67,68].

**Figure 2:**
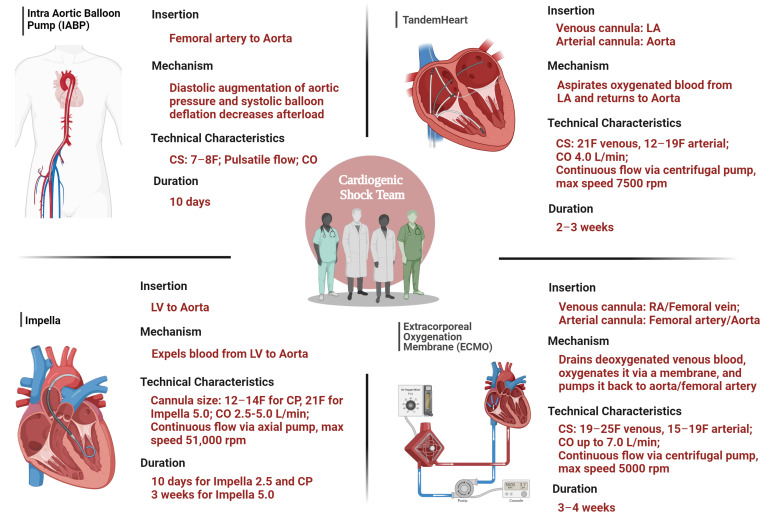
Key features that the cardiogenic shock team must consider to provide appropriate temporary mechanical circulatory support systems for each clinical scenario. Created with BioRender.com. CS: cannula size, CO, cardiac output; LA, left atrium, LV, left ventricle.

Rather than viewing MCS devices as competing technologies, it is crucial to understand the unique attributes of each device (Figure 2) to maximize their benefits in various clinical scenarios. An individualized approach, tailored to the specific needs and clinical context of each patient, is essential. In patients with valvular disease, MCS should be tailored to the specific pathology, with Impella preferred in those with severe MR, while being contraindicated in severe AS, and peripheral VA-ECMO contraindicated in AR [18,69]. For patients experiencing fulminant myocarditis, the cardiogenic shock team should prioritize biventricular support (such as VA-ECMO combined with Impella or a biventricular assist device) due to diffuse myocardial involvement, potentially necessitating long-term MCS or transplantation if myocardial recovery proves insufficient [70]. Moreover, in cases with predominant RV failure unresponsive to inotropes/vasopressors, VA-ECMO or Impella RP may be considered, especially post-LVAD implantation where right-sided mechanical support (Impella RP or Protek Duo) is needed, taking care with VA-ECMO due its effect on LVAD physiology [69,71]. A meta-analysis of four randomized clinical trials comparing early routine use of VA-ECMO versus optimal medical therapy alone in patients with infarct-related CS found no significant reduction in 30-day mortality with early VA-ECMO use [72]. In the clinical scenario of STEMI complicated by CS, the DANGER Shock trial was the first to document the clear superiority of implementing a microaxial flow pump (IMPELLA CP) over standard care [44]. These developments, along with results from pending trials, are expected to influence future guidelines and how physicians utilize MCS.

An additional important issue to understand is the pathophysiology of ventricle subjected to circulatory support, which exhibits distinct characteristics compared to a native ventricle with reduced ejection fraction [73]. In a native heart, blood flow follows a well-defined pattern from the mitral valve toward the apex and then redirects to the aortic valve, maintaining uniform kinetic energy distribution [74]. However, under circulatory support, flow dynamics are altered due to mechanical intervention, resulting in decreased flow uniformity and significant slowing at the apical site. These changes affect ventricular mechanics throughout the cardiac cycle, potentially reducing wall stress, particularly in the apical regions [75]. Understanding these altered hemodynamic patterns is essential for optimizing patient outcomes and exploring potential recovery strategies. Continuous monitoring and tailored adjustments of the support device are crucial for facilitating ventricular recovery, even though routine intraventricular mechanical analysis is not commonly performed in clinical practice. Addressing these pathophysiological differences is critical for achieving better clinical results and considering device explantation when feasible.

To optimize outcomes in the management of temporary MCS in AHF, clinicians should focus on key aspects including optimizing medications, ensuring proper anticoagulation, and maintaining vascular access to prevent complications. Regular laboratory assessments are essential for monitoring renal function, lactate levels, and hemoglobin. Imaging, especially echocardiography, is crucial for adjusting MCS support and guiding the weaning process. It provides real-time insights into cardiac function, ventricular loading, and intracardiac pressures [76]. Advanced echocardiographic parameters, such as strain imaging and three-dimensional echocardiography, offer detailed evaluations of cardiac mechanics, aiding in precise support adjustments [77]. Future perspectives on MCS management involve the integration of advanced echocardiographic techniques. These methods, including speckle-tracking echocardiography and myocardial deformation imaging, offer deeper insights into myocardial function, potentially leading to more tailored support strategies and improved patient outcomes [78].

Another concern that clinicians should keep in mind before choosing between the available temporary MCS devices is the possible complications that may arise, as these may impact patient survival. For instance, early experience with the Impella 5.5 device indicated that over one-third of patients required transfusions due to bleeding, which could lead to sensitization for those on the waitlist [79]. VA-ECMO is linked to bleeding, thrombosis, and high waitlist mortality. However, studies to date indicate that patients bridged to transplant with temporary MCS have excellent survival rates, comparable to those of non-bridged recipients [80]. Notably, recipients bridged with ECMO have shown significant improvements in post-transplant outcomes following the 2018 policy revision [81,82].

Other factors influencing the use of MCS in CS include socioeconomic status (SS) and center expertise. A recent study evaluated the impact of SS on the utilization of MCS devices and outcomes in CS. The study analyzed 38,520 hospitalizations due to CS from the State Inpatient Databases of nine U.S. states in 2016, categorizing patients into SS cohorts based on the median household income of their residential zip codes. Findings revealed that the utilization of temporary MCS devices was more prevalent in higher SES regions (21.3% in the lowest SES quartile to 24.1% in the highest SES quartile, *p* < 0.01). Interestingly, despite increased hospital costs in higher SS areas, there was no significant difference in overall mortality from CS among the different SS cohorts [83].

Furthermore, there is a substantial learning curve associated with the use of different MCS devices. Institutions with more experience tend to report better outcomes, indicating that operator skill and institutional expertise are critical to the success of these interventions. A study from Brazil by Scholari et al. involving 49 patients with CS treated with Impella CP or VA-ECMO found that the overall mortality rate decreased significantly from 83% in the initial period to 40% in the later period (*p* = 0.002). The learning curve analysis showed improved survival after 40 consecutive cases [84]. This underscores that proficiency gained through experience is essential, can significantly impact patient survival in heart failure, and may affect the results of multicenter trials in diverse populations.

Looking forward, the integration of artificial intelligence (AI) in temporary MCS holds promising potential to revolutionize patient management and outcomes. AI-driven predictive models can leverage real-time data from temporary MCS devices to estimate cardiac output, monitor ventricular unloading, and predict patient trajectories. These tools enable dynamic decision-making, optimize therapeutic protocols, and potentially automate support adjustments [85]. The development of “digital twins”—virtual replicas of patients—could simulate therapeutic scenarios, aiding in the selection of effective temporary MCS strategies, especially in complex multidevice cases [86]. Furthermore, AI algorithms can predict and manage complications such as hemocompatibility issues and assist in early stratification of patients based on their likelihood of native heart recovery [87]. Despite challenges like the need for high-quality data and robust validation, AI’s integration into temporary MCS promises personalized, adaptive care and improved clinical outcomes.

## 4. Conclusions

The role of temporary MCS in patients with advanced HF as a bridge to transplantation is multifaceted and essential. Instead of viewing these devices as rival technologies, it is crucial to comprehend each device distinctly to capitalize on their unique attributes in various clinical scenarios. Future advancements in MCS technology should focus on developing intelligent pumps capable of monitoring hemodynamics and predicting potential instability for proactive intervention. The integration of artificial intelligence (AI)-based software algorithms presents a promising avenue, as these can assist in determining the optimal support strategy—whether drug-based, device-based, or a combination—tailored to the specific needs of each patient.

Overall, the evolution of MCS devices and strategies must continue to prioritize individualized patient care, leveraging technological advancements to improve outcomes and reduce complications. Through a nuanced understanding of each device’s strengths and limitations, clinicians can better navigate the complexities of MCS and enhance the quality of care for patients with severe cardiac conditions.

## Figures and Tables

**Table 1:** Summary of trials on IABP use in Hypoperfused ADHF.

Study	Study Type	Sample Size	Patient Characteristics	IABP Insertion/Duration	Key Hemodynamic Measurements	Outcomes
Umakanthan et al. [24]	Retrospective	18	- End-stage HF - Failure/intolerance to inotropes	Axillary, 27 ± 18 days	CI, CVP, mPAP, sPAP	* Recovery: 0%, HT: 72%, Death: 28% (6% despite LVAD), 1-month survival: 89%, 6-month survival: 72%
Mizuno et al. [25]	Prospective	123 vs. 4678	ADHF, modified Framingham HF criteria, IABP vs. no IABP	Femoral, n/a	N/A	Recovery: 71%, Death: 29%
Ntalianis et al. [26]	Prospective	15	End-stage HF, NYHA IV, INTERMACS 1-2, severe LV and RV systolic dysfunction	Femoral, 73 ± 50 days	CI, mPAP, PCWP, RAP, RVSWI, creatinine, bilirubin, LVEF, RVEDD	Recovery: 20%, LVAD: 40%, Death: 40%
den Uil et al. [27]	Retrospective	27	Inotrope-dependent HF, hypoperfusion, INTERMACS 1–2	Femoral, median 4 days	MAP, RAP, SvcO2, fluid balance, sodium, serum lactate	Recovery: 26%, LVAD: 19%, Death: 26%, HT: 22%, 30 d survival: 67%, 1-year survival: 63%
Annamalai et al. [28]	Prospective	10	Stage D HF, NYHA III-IV, INTERMACS 2–3, low CO post-LVAD surgery	Femoral, <48 h	CPO, DPTI, LVESP, LVSW, PAP, PVR, myocardial oxygen supply/demand	LVAD: 100%
Hsu et al. [6]	Prospective	74	CS (89% HFrEF), SBP < 90 mm Hg, poor end-organ perfusion	Femoral, n/a	CI, DBP, HR, LVCPI, PAP, PCWP, RAP, SBP, SVR	Recovery: 20%, LVAD: 45%, Death: 24%, HT: 8%, MCS: 4%
Morici et al. [29]	Prospective	17	Severe LV dysfunction, SBP < 90 mm Hg, MAP < 60 mm Hg, RAP > 12 mm Hg	Femoral, median 7 days	N/A	Recovery: 12%, LVAD: 53%, Death: 18%, HT: 12%, ECMO: 6%
Fried et al. [30]	Retrospective	132	ADHF with CS, CI < 2.2 L/min/m^2^, SBP < 90 mm Hg	Femoral (1 axillary), median 96 h	CI, CO, mPAP	Recovery: 16%, LVAD: 52%, Death: 18%, HT: 6%, 30 d survival: 84%, MCS: 8%
Imamura et al. [8]	Retrospective	91	Advanced worsening HF (69% on inotropes)	Subclavian, 25 ± 20 days	CI, CVP, PCWP, creatinine, serum lactate	Recovery: 12%, LVAD/HT: 69%, Death: 9%, MCS: 4%
Malick et al. [5]	Retrospective	132	ADHF with CS, CI < 2.2 L/min/m^2^, SBP < 90 mm Hg	Femoral, median 3 days	CI, CO, CPI, CPO, CVP, SVR, mPAP	Recovery: 16%, HT: 62%, Death: 22%, MCS: 8%
den Uil et al. [23]	Randomized	32	Diuretic-resistant ADHF, no ischemia, IABP vs. inotropes (enoximone/dobutamine)	Femoral, median 4 days	Greater 3 h SvO2 increase in IABP group	HT: 31% (IABP) vs. 0% (inotropes), 30 d mortality: 23% (IABP) vs. 44% (inotropes)

Abbreviations: ADHF, Acute Decompensated Heart Failure; CI, Cardiac Index; CO, Cardiac Output; CPI, Cardiac Power Index; CPO, Cardiac Power Output; CS, Cardiogenic Shock; CVP, Central Venous Pressure; DBP, Diastolic Blood Pressure; DPTI, Diastolic Pressure–Time Index; ECMO, Extracorporeal Membrane Oxygenation; HF, Heart Failure; HFrEF, Heart Failure with Reduced Ejection Fraction; HT, Heart Transplant; IABP, Intra-Aortic Balloon Pump; INTERMACS, Interagency Registry for Mechanically Assisted Circulatory Support; LV, Left Ventricular; LVCPI, Left Ventricular Cardiac Power Index; LVEF, Left Ventricular Ejection Fraction; LVESP, Left Ventricular End-Systolic Pressure; LVSW, Left Ventricular Stroke Work; MAP, Mean Arterial Pressure; MCS, Mechanical Circulatory Support; mPAP, Mean Pulmonary Artery Pressure; N/A, Not available; NYHA, New York Heart Association; PAP, Pulmonary Arterial Pressure; PCWP, Pulmonary Capillary Wedge Pressure; PVR, Pulmonary Vascular Resistance; RAP, Right Atrial Pressure; RV, Right Ventricle; RVEDD, Right Ventricular End-Diastolic Diameter; RVSWI, Right Ventricular Stroke Work Index; SBP, Systolic Blood Pressure; sPAP, Systolic Pulmonary Artery Pressure; SVR, Systemic Vascular Resistance; SvcO2, Central Venous Oxygen Saturation; NTproBNP, N-terminal pro-B-type Natriuretic Peptide. * Recovery: correlated to the percentage of heart replacement therapies—free survival.

**Table 2:** Summary of studies on TH and ECMO use in ADHF.

Study	Device	Sample Size	Patient Characteristics	Duration	Key Hemodynamic Measurements	Outcomes
Smith et al. [31]	TH	18	Refractory CS	5.2 days	CI, PCWP	Survival to discharge: 23.8% (bridge to recovery) vs. 68.2% (bridge to LVAD)
Kar et al. [32]	TH	117	Refractory CS	5.8 days	CI, PCWP, SvO2, lactate, creatinine	30-day mortality: 40.2% 6-month mortality: 45.3%
ECMO-CS [33]	ECMO	117 (35.1% non-MI)	Rapidly deteriorating patients or with established CS randomized to early VA-ECMO or initially conservative therapy	N/R	N/R	No difference in composite endpoint (71.4% vs. 77.3%) or all-cause mortality (52.4% vs. 54.5%)

Abbreviations: TH, Tandemheart; ECMO, extracorporeal membranous oxygenation; ADHF, Acute Decompensated Heart Failure; CS, cardiogenic shock; CI, cardiac index; PCWP, pulmonary capillary wedge pressure; LVAD, left ventricular assist device.

**Table 3:** Summary of trials on Impella use in patients with ADHF.

Study	Study Type	Sample Size	Patient Characteristics	Impella Type/Insertion/Duration	Key Hemodynamic Measurements	Outcomes
Lemaire et al. [41]	Retrospective	47	- CS (CI < 2.2 L/min/m^2^) - 6% for ADHF	Impella 5.0 (78%)/ 83% via end-to-side anastomosis/ 5.4 ± 4.5 days	N/M	30-day mortality: 72.3%, 90-day mortality: 65.9% 1-year survival: 63.8%
Lima et al. [42]	Retrospective	40	- End-stage ADHF	Impella 5.0/ 75% via Axillary artery/ 7 ± 5 days	CI, CPO, SBP, MAP, PADP	Survival to next therapy: 75% (bridge to HT—to LVAD)
Hall et al. [39]	Retrospective	58	- End-stage ADHF - Failure/intolerance to inotropes	Impella 5.0/ 74% via Axillary artery/ 7 ± 5 days	CI, CVP, SBP, MAP, mPAP, sPAP, PCWP	Survival to next therapy: 67% 1-year survival rates: 65% (durable MCS), 87% (HT), 75% (weaned)
Kuso et al. [43]	Retrospective	194	- ADHF patients undergoing VT ablation in - MCS vs. non-MCS - Frequent electrical storm in MCS (49%)	Impella 2.5 (73%)/ 74% via Axillary artery/N/M	N/M	Death, HT and recurrent VT: 36% (MCS) vs. 26% (non-MCS)
Haddad et al. [40]	Retrospective	16	- Advanced HF patients, Impella 5.5 before HT	Impella 5.5/ Axillary artery/N/M	sPAP, PADP, PCWP, PVR	All patients survived post-HT without significant morbidity Improved renal function (creatinine: 1.55 mg/dL to 1.25 mg/dL, *p* = 0.007), increased pulmonary artery pulsatility index (2.56 to 4.2, *p* = 0.048), enhanced RV function (*p* = 0.003)
Moller et al. [44]	Randomized	355	- Patients with STEMI and cardiogenic shock, Median LVEF ~25% -Impella CP + Standard-of-care vs. Standard-of-care	Impella CP/ N/M/N/M	N/M	Lower risk of death at 180 days with MCS (45.8% vs. 58.5%, HR 0.74, *p* = 0.04); Higher need for renal-replacement therapy (41.9% vs. 26.7%, RR 1.98)

Abbreviations: ADHF, Acute Decompensated Heart Failure; CI, Cardiac Index; CO, Cardiac Output; CPI, Cardiac Power Index; CPO, Cardiac Power Output; CS, Cardiogenic Shock; CVP, Central Venous Pressure; DBP, Diastolic Blood Pressure; HF, Heart Failure; HT, Heart Transplant; LV, Left Ventricular; LVEF, Left Ventricular Ejection Fraction; MAP, Mean Arterial Pressure; MCS, Mechanical Circulatory Support; PADP, Pulmonary Artery Diastolic Pressure; PAP, Pulmonary Arterial Pressure; PCWP, Pulmonary Capillary Wedge Pressure; PVR, Pulmonary Vascular Resistance; RV, Right Ventricle; SBP, Systolic Blood Pressure; SPAP, Systolic Pulmonary Artery Pressure; SVR, Systemic Vascular Resistance; VT, Ventricular Tachycardia.

## Data Availability

Not applicable.
